# The tumor-stromal ratio as a strong prognosticator for advanced gastric cancer patients: proposal of a new TSNM staging system

**DOI:** 10.1007/s00535-017-1379-1

**Published:** 2017-08-16

**Authors:** Chunwei Peng, Jiuyang Liu, Guifang Yang, Yan Li

**Affiliations:** 1 Department of Oncology, Zhongnan Hospital of Wuhan University, Hubei Key Laboratory of Tumor Biological Behaviors and Hubei Cancer Clinical Study Center, No. 169 Donghu Road, Wuchang District, Wuhan, People’s Republic of China; 2 Department of Pathology, Zhongnan Hospital of Wuhan University, No. 169 Donghu Road, Wuchang District, Wuhan, 430071 People’s Republic of China; 3 Department of Peritoneal Cancer Surgery, Cancer Center of Beijing Shijitan Hospital Affiliated to the Capital Medical University, No. 10 Tieyi Road, Yangfangdian, Beijing, 100038 People’s Republic of China

**Keywords:** Gastric cancer, Prognosis, Tumor microenvironment, Staging system

## Abstract

**Background:**

Insufficient attention is paid to the underlying tumor microenvironment (TME) evolution, that resulting in tumor heterogeneity and driving differences in cancer aggressiveness and treatment outcomes. The morphological evaluation of the proportion of the stroma at the most invasive part of primary tumor (tumor-stromal ratio, TSR) in cancer is gaining momentum as evidence strengthens for the clinical relevance.

**Methods:**

Tissue samples from the most invasive part of the primary gastric cancer (GC) of 494 patients were analyzed for their TSR, and a new TSNM (tumor-stromal node metastasis) staging system based on patho-biological behaviors was established and assessed.

**Results:**

TSR is a new and strong independent prognostic factor for GC patients. The likelihood of tumor invasion is increased significantly for patients in the stromal-high subgroup compared to those in the stromal-low subgroup (*P* = 0.011). The discrimination ability of TSR was not less than the TNM staging system and was better in patients with stages I and II GC. We integrated the TSR parameter into the TNM staging system and proposed a new TSNM staging system creatively. There were three new subgroups (IC, IIC, IIID). There were four major groups and 10 subgroups in the TSNM system. The difference in overall survival (OS) was statistically significant among all TSNM system (*P* < 0.005 for all). Deep analyses revealed well predictive performance of the TSNM (*P* < 0.001).

**Conclusions:**

This study confirms the TSR as a TME prognostic factor for GC. TSR is a candidate TME parameter that could easily be implemented in routine pathology diagnostics, and the TSNM staging system has been established to optimize risk stratification for GC. The value of the TSNM staging system should be validated in further prospective study.

**Electronic supplementary material:**

The online version of this article (doi:10.1007/s00535-017-1379-1) contains supplementary material, which is available to authorized users.

## Introduction

Recent figures show that gastric cancer (GC) is now the second commonest cause of cancer related mortality in China with over 498,000 deaths in 2015 [1]. Despite significant advances in GC early diagnosis, staging system, and treatment, the 5-year overall survival (OS) of GC remains around 40% [2, 3]. One of the key causes is that GC is a heterogeneous disease in its biologic behaviors, but current prognostic and predictive factors for GC do not provide optimal risk stratification, thus additional information is necessary to improve tailored treatment for the individual patient [4, 5]. How to establish clinically relevant subtypes that would encompass this heterogeneity and provide useful clinical information has been considered [6]. Although many new and modified subtypes have been proposed, a tumor-based TNM staging system is still the dominant instrument to guide treatment strategy for GC patients, and insufficient attention is paid to the underlying tumor microenvironment (TME) evolution, which is the underlying cause for tumor heterogeneity, divergent cancer aggressiveness, and clinical outcomes [7, 8].

The TME concept considers the interplay between cancer cells and stromal cells as the primary driving force for cancer progression. In the past, tumor cells have been the main target for therapeutic interventions [9]. Nowadays, however, accumulating evidence illustrates that tumor stromal, as an integral component of TME, could facilitate tumor progression [10]. Some essential components of stroma, including tumor associated macrophages (TAM), cancer associated fibroblasts (CAF), and tumor-infiltrating lymphocytes (TIL) [11–13], have been evaluated. Moreover, some critical events have also been investigated and applied in clinical setting, such as targeting the immune cells and angiogenesis [14, 15]. The novel concept that the TME is built through rate-limiting steps during multistage carcinogenesis has been established. Thus, TME represents a unique tumor property and a robust target for cancer treatment and prevention [16]. However, considering the complexity components of TME, overall consideration about the evolution of stromal should be highlighted [17, 18].

The morphological evaluation of the ratio of stromal at the most invasive part of primary tumor (tumor-stromal ratio, TSR) is gaining momentum as evidence strengthens for the clinical relevance of this TME parameter. TSR was first reported as an independent stromal parameter for survival as compared with lymph node status and tumor stage in colon cancer, and there was a preliminary attempt to adding the stromal parameter to the ASCO colon risk criteria [19]. It was concluded that TSR should be integrated into the standard pathology reports in addition to the current TNM classification [20]. As a newly identified prognostic factor, it was also found to be significantly associated with prognosis of esophageal squamous cell carcinoma, breast cancer, and cervical cancer [21–23]. Accumulating evidence suggests that the important role of TME could be generally assessed by the TSR evaluation of simple hematoxylin and eosin (H&E)-stained tumor sections [19].

With these considerations in mind, we hypothesize that TSR would be a suitable TME parameter for GC that may parallel the evolving tumor-stromal interactions. We have undertaken a comprehensive analysis of the TME of GC, and evaluated the prognostic and predictive role of TSR in GC, and stratify the GC patients into four groups based on TNM staging system and TSR. TSR could be easily implemented in routine daily pathology diagnostics, as it is simple to determine, reproducible, and performed in a short time.

## Materials and methods

### Study population and database

The records of patients who underwent surgical resection of GC from December 2002 to February 2011 were reviewed. Major demographic and clinicopathological characteristics were retrieved. The tumor type, histologic grade, depth of invasion, number of lymph nodes retrieved, and number of lymph nodes with metastases were re-confirmed histologically. Inclusion and exclusion criteria were defined as follows. Patients were included when histology confirmed adenocarcinoma of the stomach and the survival data were available. Patients were excluded when distant metastasis has been diagnosed before surgery, histology identified a pathological type other than adenocarcinoma, R1 resection, no lymph node was retrieved or histopathological and survival data were incomplete. No patients receive neoadjuvant chemotherapy. In this study, 329 (66.6%) patients received adjuvant chemotherapy. In stage III patients (*n* = 316), 217 (68.7%) patients received adjuvant chemotherapy, and only 69 patients received more than six cycles of adjuvant chemotherapy. TNM stage was determined according to the 7th edition UICC/AJCC TNM system. Overall survival (OS), defined as the duration from operation to death or last follow-up, was used for prognosis evaluation. The primary endpoint of this study was OS, and patients alive at the last follow-up were recorded as censored events.

### Ethics statement

All patients provided written informed consent for their information to be stored in the hospital database, and we obtained separate consent for use of research. Study approval was obtained from independent ethics committees from Zhongnan Hospital of Wuhan University. The study was undertaken in accordance with the ethical standards of the World Medical Association Declaration of Helsinki.

### Gastric cancer specimens and histopathological protocol

**Fig. 1:**
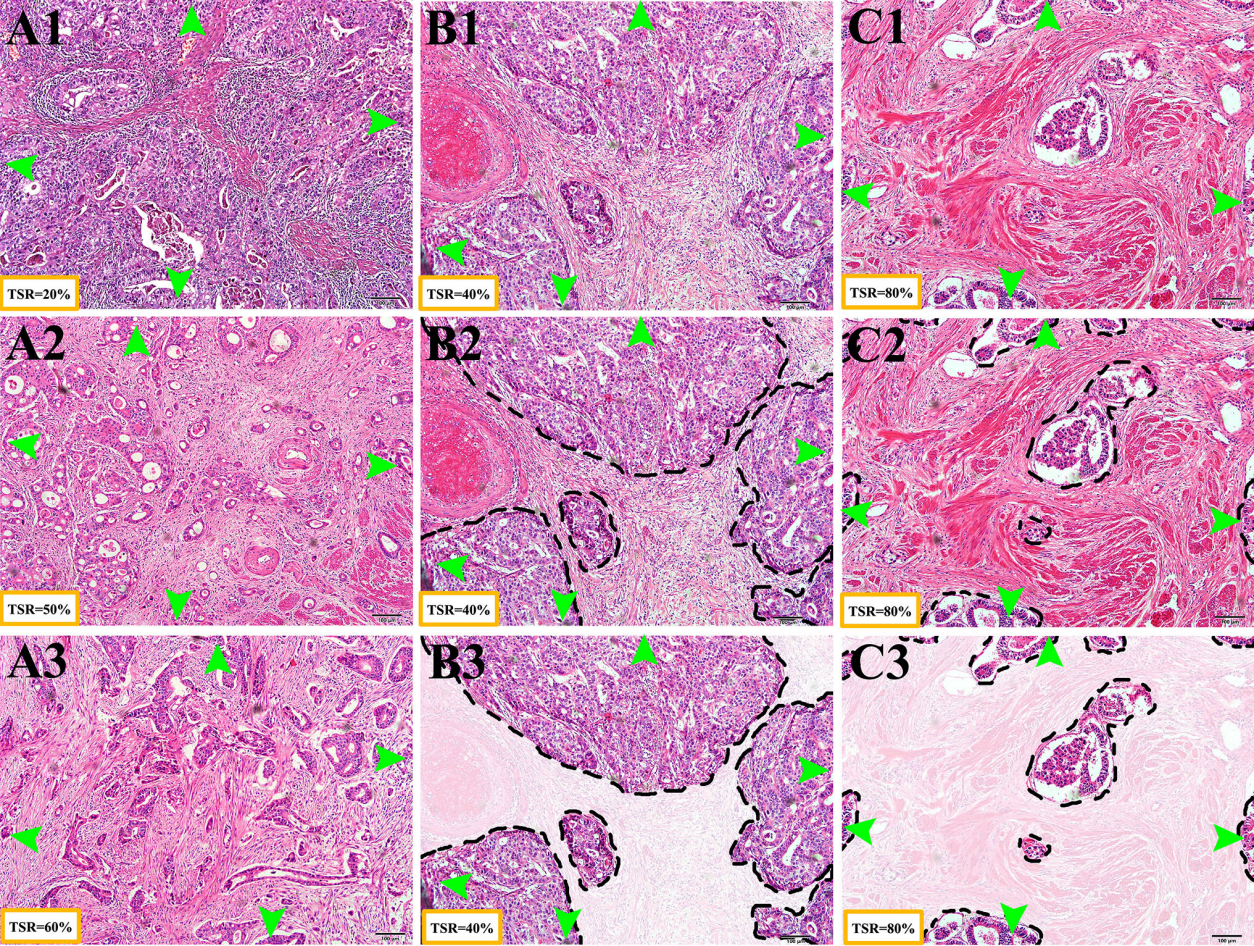
The assessment of TSR. Haematoxylin and eosin (H&E) stained 4 μm paraffin sections were examined of the most invasive part of primary GC. Tumor cells must be present at all borders of the image field (north–east–south–west, *green arrows*). **a** Typical example of TSR. (*A1*) stromal-low (TSR = 20%), (*A2*) stromal-high (50%), and (*A3*) stromal-high (60%). **b** Guideline for TSR assessment (stromal-low, TSR = 40%). (*B1*) select field, (*B2*) define stromal area, (*B3*) assess the TSR. **c** Guideline for TSR assessment (stromal-low, TSR = 80%). (*C1*) select field, (*C2*) define stromal area, (*C3*) assess the TSR. Please note that the *black dashed line* indicates the boundary of cancer nests, and the stromal were lighted graphically

The methods were performed as published [19, 21]. All H&E-stained sections were reexamined by independent reviewers who were not aware of the clinical characteristics or clinical outcomes. Tissue samples consisting of 4 μm H&E-stained sections from the most invasive part of the primary tumor were used for analysis using conventional microscopy. The invasive front was chosen from the tissue block the pathologist selected as the most invasive part and this section was used to determine the T-status. The most invasive tumor area on each slide was selected using a 2.5× or 5× objective. From this section, the TSR was visually estimated on the basis of morphological characteristics using a 10× objective. Stromal tissue not containing any tumor cells was considered not to have an apparent evolution. Thus, tumor cells must be present at all borders of the image field (north–east–south–west) (Fig. 1). Briefly, the boundaries of the invasive tumor are identified with stromal cells inside them. Stromal cells in areas with crush artifacts, necrosis, and inflammation around biopsy sites or extensive central regressive hyalinization are not scored. A necrotic biopsy is considered unscorable. When mucinous tissue was present within a field that matched our scoring criteria, the mucinous tissue was visually excluded for scoring. Two investigators estimated the stromal percentage in a blinded manner in five fields. In case of an inconclusive score, a third observer was decisive. Scoring percentages were given fivefold (5, 10, 15, 20% etc.) per image field. Then five data points were documented, and the TSR was calculated as the mean. The cut-off point for TSR was explored by “the best cut-off approach by log-rank test” [24], and 50% was defined as the cut-off point. Stroma ratio groups were divided into “stroma-high” (>50%) and “stroma-low” (≤50%). Representative examples of microscopic fields selected for TSR quantification from “stromal-rich” and “stromal-poor” tumors were showed in Fig. 1.

### Statistical analysis

Statistical analyses were performed with SPSS software (version 19.0, IBM SPSS). The Pearson *χ* ^2^ test or Fisher’s exact test was used to compare qualitative variables. Kaplan–Meier analysis was used for survival analysis and significance among patients’ subgroups was calculated by log rank test. The Cox regression model was used to perform multivariate analysis. Logistic regression was used to assess the influence of binary factors. Receiver operating characteristic curve (ROC) analysis was used to determine the predictive value of the parameters. Two-sided *P* < 0.05 was considered as statistically significant.

## Results

### Study population and the relationship between TSR and clinicopathological characteristics

A total of 494 eligible patients including 349 (70.6%) men and 145 (29.4%) women were enrolled, with age ranging from 20 to 85 (median 59) years. There were 27 (5.5%), 77 (15.6%), two (0.4%), 292 (59.1%), and 96 (19.4%) patients with T1, T2, T3, T4a, and T4b grade, respectively. No patients with distant metastasis were included, and there were 68 (13.8%), 110 (22.2%), and 316 (64.0%) patients with stage I, stage II, and stage III, respectively. According to the Japanese classification of gastric carcinoma (3rd English edition) [25], there were 136 (27.5%), 131 (26.5), 197 (39.9%), and 30 (6.1%) patients with tumors in the upper, middle, lower, and whole stomach, respectively. Major clinicopathological characteristics were listed in Table 1.

**Table 1:** Basic Characteristics

Items	Total *N* (%)	Stromal-low (TSR < 0.5) *N* (%)	Stromal-high (TSR ≥ 0.5) *N* (%)	*P* value^a^
Age in years (*M* ± SD)	59.0 ± 11.9	58.8 ± 12.1	59.1 ± 11.7	0.604
Gender				
Male	349 (70.6)	183 (72.0)	166 (69.2)	0.491
Female	145 (29.4)	71 (28.0)	74 (30.8)	
Tumor location^b^				
Upper	136 (27.5)	77 (30.3)	59 (24.6)	0.260
Middle	131 (26.5)	69 (27.2)	62 (25.8)	
Low	197 (39.9)	91 (35.8)	106 (44.2)	
All	30 (6.1)	17 (6.7)	13 (5.4)	
Pathological types				
Adenocarcinoma (Histological grade 1/2, well/moderately differentiated)	130 (26.5)	60 (23.9)	70 (29.3)	0.480
Adenocarcinoma (Histological grade 3/4, low/undifferentiated)	281 (57.3)	146 (58.2)	135 (56.5)	
Mucinous adenocarcinoma/signet-ring cell carcinoma	66 (13.5)	38 (15.1)	28 (11.7)	
Others	13 (2.7)	7 (2.8)	6 (2.5)	
pT status				
T1/T2	104 (21.1)	65 (25.6)	36 (16.3)	**0.011**
T3/T4	390 (78.9)	189 (74.4)	201 (83.8)	
pN status				
N0	166 (33.6)	88 (34.6)	78 (32.5)	0.620
N1	98 (19.8)	51 (20.1)	47 (19.6)	
N2	121 (24.5)	65 (25.6)	56 (23.3)	
N3	109 (22.1)	50 (19.7)	59 (24.6)	
TNM stages				
I	68 (13.8)	41 (16.1)	27 (11.3)	**0.086**
II	110 (22.2)	62 (24.4)	48 (20.0)	
III	316 (64.0)	151 (59.4)	165 (68.8)	
I/II	178 (36.0)	103 (40.6)	75 (31.3)	**0.031**
III	316 (64.0)	151 (59.4)	165 (68.8)	

*OS* overall survival, *TSR* tumor stromal ratio

^a^ Bold indicates values with a significant difference *P* < 0.05

^b^ Tumor location were classified according to the Japanese classification of gastric carcinoma (3rd English edition)

Estimation of the TSR was performed successfully in all 494 eligible patients. The median TSR were 0.4800 and the mean of TSR was 0.4803. Cut-off point for TSR was explored by “the best cut-off approach by log-rank test”, and the cut-off point was 0.50. That is revealed an almost perfect agreement in the cut-off points to colorectal cancer and breast cancer [19, 23]. Thus, two subgroups of TSR classification stromal-low (TSR < 0.5) and stromal high (TSR ≥ 0.5) were determined in this study (Fig. 1). Out of 494 analyzed samples, 254 (51.4%) patients were scored as stromal-low and 240 (48.6%) patients were scored as stromal-high. The TSR correlated with the clinicopathological features as summarized in Table 1. The likelihood of tumor invasion was increased significantly for stromal-high subgroup [odds ratio, OR = 1.772 (95% CI 1.137–2.763), *P* = 0.011, logistic regression]. TSR correlated with TNM stage (*P* = 0.031), but not with with age, gender, tumor location, histological grade, lymph node status, and lymph node ratio (*P* > 0.05 for all).

### Survival analysis

For 494 cases, the median OS was 27.0 (range 0.8–102.3) months, and the 1-, 3-, and 5-year survival rate was 83.4, 55.8, and 43.0%, respectively. As expected, those traditional factors were associated with GC patients’ OS, such as age, histological grade, lymph node (pN) status, serosa invasion (pT) status, and TNM stage (*P* < 0.05 for all). Although the difference between men and women was not statistically significant, women with GC seem to have poor OS (*P* = 0.068). Eighty-four (31.9%) patients in the stromal-low group and 117 (49.4%) patients in the stromal-high group died. The estimated 3- and 5-year survival rate (%) in the stromal-low group was 66.19 and 58.68%, respectively. The estimated 3- and 5-year survival rate (%) in the stromal-high group was 44.71 and 27.64%, respectively. The difference between stromal-low and stromal-high groups was statistically significant in terms of OS (*P* < 0.0001). Detailed data was showed in Table 2, and the Kaplan–Meier survival curves of OS were showed in Supplement Fig. 1.

**Table 2:** Analyses of factors regarding overall survival (OS)

Variables	*N*	No. of deaths (%)	3-year survival rate (%)	5-year survival rate (%)	Log-rank test *χ* ^2^ value	*P* value^a^
Age (years)						
<60	249	89 (35.7)	62.65	52.23	7.872	**0.005**
≥60	245	110 (44.9)	48.18	33.05		
Gender						
Male	349	136 (39.0)	58.20	46.34	3.332	0.068
Female	145	63 (43.5)	49.27	34.06		
Histological grade						
1/2	164	56 (34.1)	63.84	52.83	9.612	**0.002**
3/4	330	143 (43.3)	51.56	37.44		
Location						
Upper	136	55 (40.4)	55.11	48.53	0.04	0.842
Middle	131	56 (42.8)	52.83	39.58		
Low	197	75 (38.1)	60.47	43.46		
Total	30	13 (43.3)	42.90	42.92		
pN status						
N0	166	37 (22.3)	74.01	66.23	48.54	**<0.0001**
N1	98	41 (1,8)	55.70	41.58		
N2	121	58 (47.9)	46.14	38.04		
N3	109	63 (57.8)	41.33	16.46		
pT status						
T1/T2	104	19 (18.3)	76.09	70.84	22.606	**<0.0001**
T3/T4	390	180 (41.2)	50.77	36.38		
TNM						
I/II	178	42 (23.6)	73.66	63.75	35.073	**<0.0001**
III	316	157 (49.7)	45.86	31.81		
TSR						
Stromal-low (TSR < 0.5)	254	75 (29.5)	66.19	58.68	25.566	**<0.0001**
Stromal-high (TSR ≥ 0.5)	240	124 (51.7)	44.71	27.64		

*OS* overall survival, *TSR* tumor stromal ratio

^a^ Bold indicates values with a significant difference *P* < 0.05

### Multivariate analysis and predictive accuracy

In univariate analyses, we first studied the correlation between OS and the characteristics, both of which were included, but not included in the TNM system (age, gender, tumor histological grade, tumor location, TSR, pT status, pN status, and TNM stage) (Table 3). In multivariate analyses, all factors found to have statistically significant correlations with OS in univariate analyses were included. In this study, age, histological grade, TNM stage, and TSR were independent prognostic factors for OS after excluding other confounding factors (*P* < 0.05 for all). Compared to patients in the stromal-low group, the risk of death was increasing by 91% for patients in the stromal-high group [HR = 1.911 (95% CI 1.427–2.559), *P* < 0.0001] (Table 3).

**Table 3:** Univariate and multivariate analyses of factors associated with overall survival (OS)

Factors	Univariate analysis			Multivariate analysis		
	HR	95% CI	*P* value^a^	HR	95% CI	*P* value^a^
Age (years)						
<60	1.000			1.000		
≥60	1.489	1.125–1.971	**0.005**	1.587	1.196–2.105	**0.001**
Gender						
Male	1.000					
Female	1.320	0.979–1.781	0.069			
Histological grade						
1/2	1.000					
3/4	1.624	1.192–2.214	**0.002**	1.537	1.114–2.120	**0.009**
Location						
Upper	1.000					
Middle	1.254	0.864––1.820	0.233			
Low	0.957	0.676–1.356	0.805			
Total	1.497	0.817–2.743	0.191			
pN status						
N0 (N negative)	1.000					
N1–3 (N positive)	2.630	1.840–3.761	**<0.0001**			0.795
pT status						
T1/T2	1.000					
T3/T4	2.984	1.859–4.790	**<0.0001**			0.156
TNM						
I/II	1.000			1.000		
III	2.984	1.859–4.790	**<0.000**1	2.239	1.574–3.185	**<0.0001**
TSR						
Stromal-low (TSR < 0.5)	1.000			1.000		
Stromal-high (TSR ≥ 0.5)	2.066	1.550–2.755	**<0.0001**	1.911	1.427–2.559	**<0.0001**

*OS* overall survival, *LNR* lymph node ratio, *TSR* tumor stromal ratio

^a^ Bold indicates values with a significant difference *P* < 0.05. HR and 95% CI were not provided when *P* ≥ 0.05

Predictive value of the four independent factors was further studied by ROC analysis. Among the tested factors, histological grade was the weakest risk factor for death, and TSR performed well in predicting the clinical outcomes of GC patients compared to other factors [Area under the curve: 0.615 (95% CI 0.584–0.665), *P* < 0.001] (Supplement Fig. 1). All results have showed that TSR is a strong independent factor for GC prognosis.

### Discrimination ability of TSR in TNM staging system

Give the results abovementioned, TSR is considered to represent a strong independent TME parameter for GC prediction and prognosis. It is termed pS (pathological stromal status) in this study. The pS indicates the status of TSR, and S0 indicates stromal-low (TSR < 0.5), S1 indicates stromal-high (TSR ≥ 0.5). Further analyses were focused on the discrimination ability of TSR to identify GC patients in high risk. Traditional TNM staging system is heterogeneous, and the estimated 3- and 5-year survival rate of each TNM stage are shown in Supplement Table 1. When classified into three major groups, TNM staging systems showed good discrimination power among stages I through III (*P* < 0.0001 for all, stage I vs. stage II, *P* = 0.004; stage I vs. stage III, *P* < 0.0001; stage II vs. stage III, *P* < 0.0001).

**Fig. 2:**
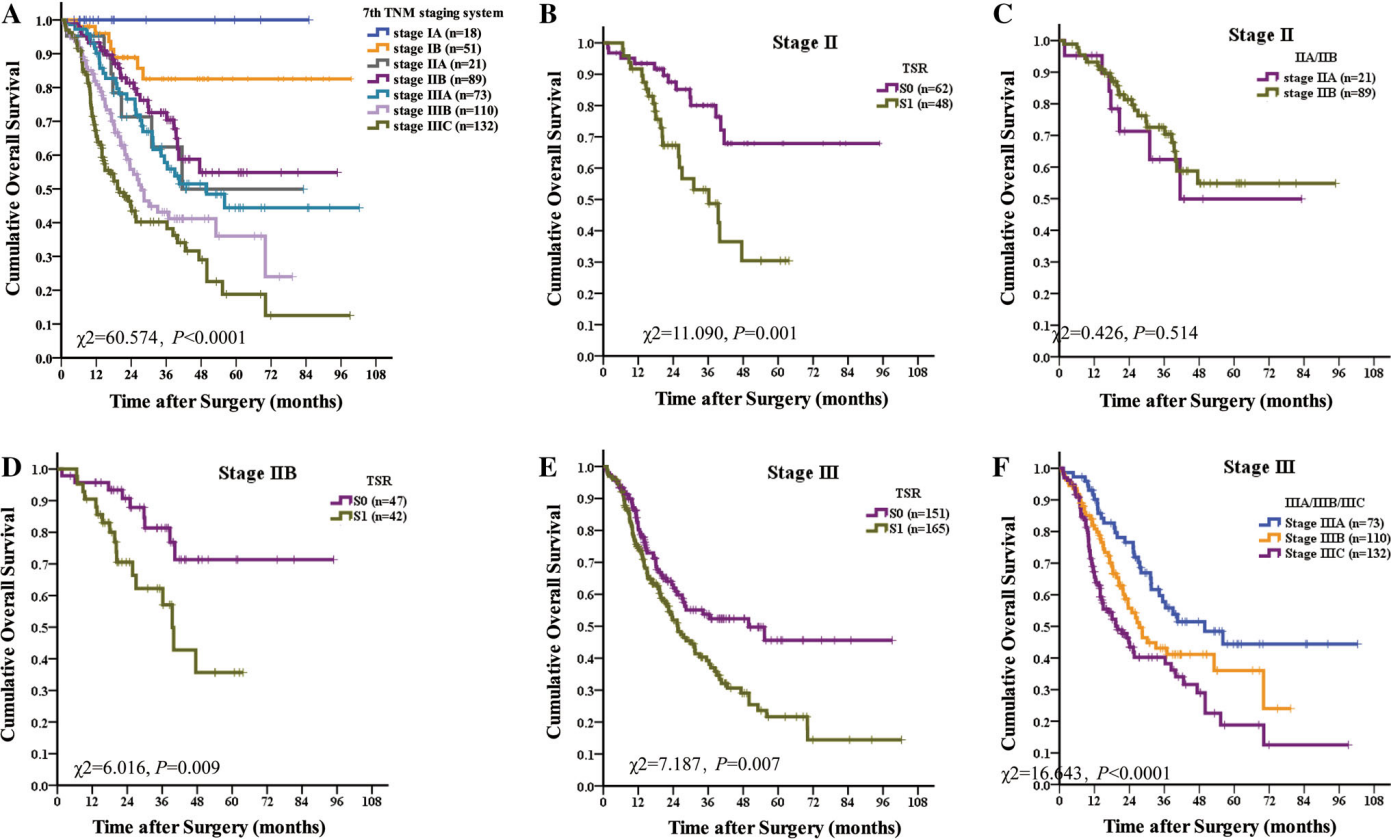
Discrimination ability of TSR in the TNM staging system. **a** TNM staging systems are heterogeneous. **b** Among stage II, there was statistically significant separation between the stromal-low group and the stromal high group (*P* = 0.001). **c** Among stage II there was no statistically significant separation between stages IIA and IIB (*P* = 0.514). **d** Among stage IIB, TSR showed strong discrimination ability. **e** Among stage III, there was statistically significant separation between the stromal-low group and the stromal high group (*P* = 0.007). **f** Among stage III there was statistically significant separation among stages IIIA, IIB, and IIIC (*P* < 0.0001)

Group analyses were performed among each stage of TNM system in terms of TNM stage and TSR to show the discrimination ability of TSR. Among stage I, there was no statistically significant separation between stages IA and IB (*P* = 0.227), and there was no statistically significant separation between the S0 group and the S1 group (*P* = 0.123). Among stage II, there was no statistically significant separation between stages IIA and IIB (*P* = 0.514), there was statistically significant separation between the S0 group and the S1 group (*P* = 0.001). Among stage III, there was statistically significant separation among stages IIIA, IIB, and IIIC (*P* < 0.0001), there was statistically significant separation between the S0 group and the S1 group (*P* = 0.007). The discrimination ability of TSR was not less than the TNM staging system and was better in patients with stages I and II (Supplement Table 1; Fig. 2).

When classified into seven stages, there were 1, 2, 3, 4, 3, 4, 3 subgroups in stage IA, IB, IIA, IIB, IIIA, IIIB, and IIIC. And the TNM staging systems was homogeneous in each stage, although patients were heterogeneous, and those with poor prognosis in subgroups should be identified (Table 4). TSR showed strong discrimination ability in stages IIA and IIB (*P* < 0.05 for all).

**Table 4:** The distribution of the study population

TNM stage			Stromal	*N*	No. of deaths (%)	3-year survival rate	5-year survival rate	Log-rank test *χ* ^2^ value	*P* value^b^
Stage (*n*)	pT	pN	pS^a^						
IA (18)	T1	N0		18	0	100%	100%	NA	
			S0	8	0	100%	100%	NA	
			S1	10	0	100%	100%		
IB (51)	T2	N0		49	7 (14.3)	81.3%	81.3%	0.320	0.571
	T1	N1		2	0	100%	100%		
			S0	34	3 (9.8)	89.0%	89.0%	2.828	0.093
			S1	17	4 (23.5)	66.2%	66.2%		
IIA (21)	T3	N0		2	1 (50)	50.0%	0%	0.434	0.510
	T2	N1		13	5 (38.5)	55.7%	33.4%		
	T1	N2		6	1 (16.7)	75.0%	75.0%		
			S0	16	3 (18.7)	84.4%	63.3%	8.455	**0.004**
			S1	5	4 (80)	0%	0%		
IIB (89)	T4a	N0		76	21 (27.6)	74.7%	59.4%	2.500	0.114
	T3	N1		0	0	NA			
	T2	N2		12	4 (33.3)	61.1%	0%		
	T1	N3		1	1 (100)	0%	0%		
			S0	47	9 (19.1)	81.2%	71.1%	6.916	**0.009**
			S1	42	17 (40.5)	61.2%	37.9%		
IIIA (73)	T4a	N1		69	31 (44.9)	56.9%	42.7%	0.430	0.512
	T3	N2		0	0	NA			
	T2	N3		4	1 (25)	75.0%	0		
			S0	37	13 (35.1)	67.3%	55.1%	2.087	0.149
			S1	36	19 (52.8)	47.1%	31.4%		
IIIB (110)	T4b	N0		21	8 (38.1)	20.0%	20.0%	0.389	0.533
	T4b	N1		13	5 (38.5)	32.9%	0%		
	T4a	N2		76	39 (51.3)	45.6%	37.4%		
	T3	N3		0	0	NA			
			S0	47	19 (40.4)	49.2%	49.2%	2.230	0.135
			S1	63	33 (52.4)	37.8%	271%		
IIIC (132)	T4b	N2		29	15 (51.7)	36.7%	36.7%	0.346	0.556
	T4b	N3		30	18 (60)	40.1%	0%		
	T4a	N3		73	42 (57.5)	41.6%	21.1%		
			S0	65	28 (43.1)	42.9%	32.2%	2.866	0.090
			S1	67	47 (70.1)	36.6%	12.4%		

*OS* overall survival, *TSR* tumor stromal ratio, *NA* not available

^a^ pS indicates the status of TSR. TSR is considered as a represent factor of stromal, and the S0 indicates stromal-low (TSR < 0.5), the S1 indicates stromal-high (TSR ≥ 0.5)

^b^ Bold indicates values with a significant difference *P* < 0.05

### A proposal for TSNM system

**Fig. 3:**
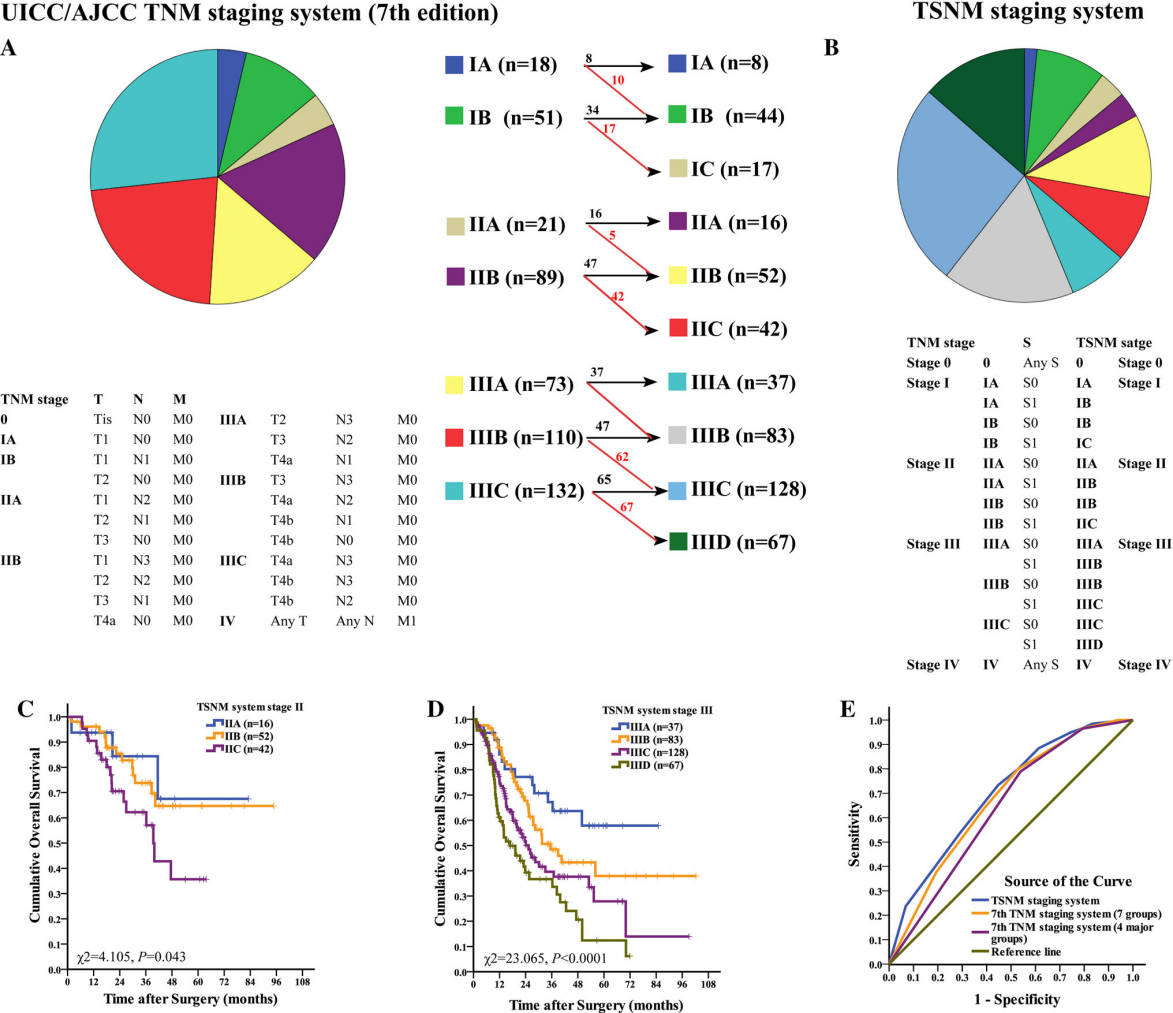
Definitions and patients distribution in the two staging systems. **a** The 7th TNM staging system. **b** The TSNM staging system, established based on 7th pT, 7th pN, 7th pM classification and pS status, the general rules for grouping were redefined. Detailed changes in stage distribution were shown in this figure. *Number* in *red* indicates patients upstaged, *number* in *black* indicates patients with the same stage. **c** Cumulative OS of GC patients in TSNM stage II. **d** Cumulative OS of GC patients in TSNM stage III. **e** TSNM performed well in predicting the clinical outcomes of GC patients compared to other factors

Sixty-four percent of patients were in stage III. Those patients with a high risk of death should be identified exactly as much as possible. All results mentioned in this paper have shown that TSR is a strong independent factor for GC prognosis, and the discrimination ability for classification to discriminate patients with poor OS was not worse than the cancer cell-based TNM staging system. The advantages of TSR matched the requirement for a factor to be included in the staging system. Thus, we proposed that factors representing the TME status can and should be included into the staging system. This new staging system based on pathobilogical behavior for GC is the TSNM (tumor-stromal node metastasis) system. The comparison between the TNM system and the TSNM system are shown in Fig. 3. Given that the four major groups in the TNM system performed very well, these groups were not changed in the TSNM system. TSR was added in subgroups. Briefly, patients in the S1 group were upstaged, and patients in the S0 group stayed in the same stage. There were three new subgroups (IC, IIC, IIID). Then, there were four major groups and 10 subgroups in the TSNM system. The difference of OS was statistically significant among all TSNM systems (*χ* ^2^ = 65.941, *P* < 0.0001), stage II (*χ* ^2^ = 4.105, *P* = 0.043), and stage III (*χ* ^2^ = 23.065, *P* < 0.0001). Moreover, subgroup analyses showed that the difference of OS was statistically significant between IB and IC, IIB and IIC, IIIB and IIIC, IIIC and IIID, IIIA and IIIC, IIIA and IIID (*P* < 0.05 for all).

Predictive value of the 7th TNM staging system (four major groups), 7th TNM staging system (seven groups) and TSNM staging system were further studied by ROC analysis. Among the tested factors, 7th TNM staging system (four major groups) was the weakest risk factor for death [area under the curve: 0.639 (95% CI 0.590–0.687), *P* < 0.0001], and TSNM [area under the curve: 0.699 (95% CI 0.653–0.745), *P* < 0.0001] were better to predict the clinical outcomes of GC patients compared to 7th TNM staging system (seven groups) [area under the curve: 0.673 (95% CI 0.626–0.720), *P* < 0.0001] (Fig. 3).

## Discussion

We have shown that TSR is a useful TME criterion for the prediction of tumor survival in patients with GC. Herein, we answered three novel questions. First, do we observe a suitable TME parameter that parallels the evolving tumor-stroma interactions? Second, is the TME parameter comparable or more powerful than the cancer cell-based histopathological criteria in predicting prognosis? And third, in which way can the TME parameter be integrated into standard pathology reports in addition to the current TNM staging system for better risk classification?

Compared to the progress in TME theory and TME-based therapy, the application of the TME concept in cancer prognosis is relatively stagnant [26, 27]. Although researchers have shown the predictive power arises from genes expressed by stromal cells [28], identifying of new convenient TME prognostic factors and integrating into the current TNM staging system may have profound influence for tumor prognosis and therapeutic strategies. Alternatively, morphometrical analysis enables accurate quantification of various tissue components when compared with qualitative systems. Here, we evaluated the TSR by pathologists. Our results indicated that TSR was a new and strong independent prognostic factor for GC. TSR was correlated with TNM stage (*P* = 0.031) and the risk of death was increasing 91% for patients in the stromal-high group compared to patients in the stromal-low group [HR = 1.911 (95% CI 1.427–2.559), *P* < 0.0001]. Receiver operating characteristic curve showed that TSR performed well in predicting the clinical outcomes of GC patients compared to other factors [area under the curve: 0.615 (95% CI 0.584–0.665), *P* < 0.001] (Fig. 2).

TSR is a robust prognostic factor in cancer, while it may be argued that to what extent the TSR could represent the stromal status. Based on the progression of TME theory, we hypothesize that evolving tumor-stromal interactions contributed to the presence and variable of TSR. Previously, we suggested that tumor cells can activate their adjacent stroma during the TME evolution process. The activated stromal, which receive and elicit oncogenic signals and undergo quantitative and morphological changes, was the source of promoting cancer progression [18, 29]. There are several theories as to why a high proportion of stromal within a tumor may infer a poor prognosis. First, it has been hypothesized that tumors with a greater proportion of stroma are able to produce more stroma-derived growth factors thus increasing the overall tumor burden [30]. Second, it has been suggested that the relative amount of desmoplastic fibrosis may have a role in reducing the accessibility of tumors to the immune response by encapsulating the malignant cells and preventing their destruction [31, 32]. More importantly, high proportion of stromal means that more stromal are activated by fewer cancer cells, indicating that the coevolution between cancer cells and tumor stromal is more effective, and consequently, results in poor overall survival. Our result showed that the likelihood of tumor invasion was increased significantly for patients in stromal-high subgroup compared to those in stromal-low group (*P* = 0.011). Our study underlined this hypothesis that an increased amount of stromal involvement, even when it is detected in only a small part of the total tumor mass, can be linked to an unfavorable prognosis independent of other prognostic parameters. Possibly, this particular part of the tumor has obtained the capability to orchestrate its direct environment to facilitate its invasive and metastatic behaviors [33, 34]. Thus, we answered the first and second novel questions that TSR would be a rational and typical TME parameter for tumor prognosis.

In addition, we integrated the TSR into TNM staging system, and proposed a new staging system creatively. This new staging system based on the pathobilogical behavior for GC is the TSNM (tumor-stromal node metastasis) system (Fig. 2), which integrates cancer cell status (invasive depth, lymph node spread, distant metastasis) and stromal behaviors (activated, inactivated). This study highlighted the importance of integrating cancer status and stromal status. Deep analysis revealed the better predictive performance of the TSNM (Fig. 2). This new staging system is a good paradigm to encompass the tumor heterogeneity and provide useful clinical information can improve current high-risk stratification methods.

Since we have shown the prognostication power, discrimination ability and availability of TSR and TSNM staging system for GC prognosis in this study, validation of convenience and reproducibility of TSR and TSNM should be performed for further application. The determination of TSR proved to be a quick and convenient procedure, which can be performed during pathological examination, using standard H&E sections without the need for additional costs above standard diagnostics. It is a simple, relatively inexpensive morphometrical measurement. To test the reproducibility, it should be emphasized that if the primary purpose is to find an approach for daily practice, the impact on daily routine should be minimal without a significant increase in the pathologist’s time [11]. Recent studies have shown that the Cohen’s kappa coefficient is high in some cancer patients, including those with breast cancer [23, 35], esophageal cancer [22], cervical cancer [21], and colorectal cancer [19, 20, 36]. These kappa values can be translated as almost in perfect agreement when evaluating TSR. Therefore, we believe that the TSR may be a candidate parameter to be implemented as a standard procedure within routine pathology laboratories. Although the results are promising, the limitations should be realized. At present, the criteria for unequivocal identification of TSR are currently unclear, and there are no established thresholds. More validation studies should be performed before the parameter can be integrated into future clinical trials, translational research, and diagnostic practice. Then, sufficient evidence would facilitate the widespread use of TSR and the TSNM staging system.

In the study of Ueno and colleagues, stromal was classified into three types based on the desmoplastic reaction (DR) resulting from stromal fibroblasts. Prognostic role of stromal type has been established [37, 38]. Given the complexity of TME, the stromal types could be defined based on the stromal cell ratio, stromal cell density, ECM density, and other parameters. However, no evidence has shown which definition of stromal types is best. In the future, we would classify stromal type deeply based on stromal cellular components (macrophages, cancer associated fibroblasts, tumor-infiltrating lymphocytes, etc.) and non-cellular components (ECM, etc.). In addition, only two patients (0.4%) with T3 tumors were included in this study such bias of distribution in populations would influence the result. In the future, multiple-institution based validation should be performed to verify the proportion of T3 tumors.

In conclusion, TSR is a convenient and useful TME parameter for oncologists to obtain more prognostic information, and the concept of integrating the TME parameter into the TNM staging system is available. TSR has shown to be a prognostic parameter and is easy to determine, is reproducible, and quickly performed for all types of GC. Because of its low cost, availability, convenience, high reproducibility, and importance in prognosis, TSR is a candidate TME parameter that could easily be implemented in routine pathology diagnostics, and TSNM staging system has been established to optimize risk stratification for GC. The value of TSNM staging system should be validated in further perspective study.

## Electronic supplementary material

Below is the link to the electronic supplementary material.

Supplement Fig. 1. Cumulative OS of GC patients. (A) OS of 494 GC patients. (B) OS of all patients in TNM staging system. (C) Patients with lymph node metastasis was related to poor OS. (D) Patients in stromal-high group were as high risk for death. (E) Patients with serosa invasion was related to poor OS. (F) TSR performed well in predicting the clinical outcomes of GC patients compared to other factors. Area under the curve for TSR was 0.615 (95%CI: 0.584-0.665, *P* < 0.001). Area under the curve for histological grade was 0.542 (95%CI: 0.491-0.594, *P* = 0.110), Area under the curve for 7^th^ TNM staging system was 0.625 (95%CI: 0.576-0.674, *P* < 0.001); Area under the curve for age was 0.528 (95%CI: 0.496-0.599, *P* = 0.073). (JPEG 3209 kb)

Supplementary material 2 (DOCX 22 kb)

## Abbreviations

GC: Gastric cancer
OS: Overall Survival
TME: Tumor microenvironment
TAM: Tumor associated macrophages
CAF: Cancer associated fibroblasts
TIL: Tumor-infiltrating lymphocytes
TSR: Tumor-stromal ratio
H&E: Hematoxylin and eosin

## References

[1] Chen W, Zheng R, Baade PD (2016). Cancer statistics in China, 2015. CA Cancer J Clin.

[2] Hartgrink HH, Jansen EPM, van Grieken NCT (2009). Gastric cancer. Lancet.

[3] Bang YJ, Kim YW, Yang HK (2012). Adjuvant capecitabine and oxaliplatin for gastric cancer after D2 gastrectomy (CLASSIC): a phase 3 open-label, randomised controlled trial. Lancet.

[4] Mlecnik B, Tosolini M, Kirilovsky A (2011). Histopathologic-based prognostic factors of colorectal cancers are associated with the state of the local immune reaction. J Clin Oncol.

[5] Peng CW, Wang LW, Zeng WJ (2013). Evaluation of the staging systems for gastric cancer. J Surg Oncol.

[6] Cristescu R, Lee J, Nebozhyn M (2015). Molecular analysis of gastric cancer identifies subtypes associated with distinct clinical outcomes. Nat Med.

[7] Jeng KS, Chang CF, Jeng WJ (2015). Heterogeneity of hepatocellular carcinoma contributes to cancer progression. Crit Rev Oncol Hematol.

[8] Quail DF, Joyce JA (2013). Microenvironmental regulation of tumor progression and metastasis. Nat Med.

[9] Hanahan D, Weinberg RA (2000). The hallmarks of cancer. Cell.

[10] Hanahan D, Weinberg RA (2011). Hallmarks of cancer: the next generation. Cell.

[11] Salgado R, Denkert C, Demaria S (2015). The evaluation of tumor-infiltrating lymphocytes (TILs) in breast cancer: recommendations by an International TILs Working Group 2014. Ann Oncol.

[12] Ruffell B, Coussens LM (2015). Macrophages and therapeutic resistance in cancer. Cancer Cell.

[13] Cirri P, Chiarugi P (2012). Cancer-associated-fibroblasts and tumour cells: a diabolic liaison driving cancer progression. Cancer Metastasis Rev..

[14] Sennino B, McDonald DM (2012). Controlling escape from angiogenesis inhibitors. Nat Rev Cancer.

[15] Buchert M, Burns CJ, Ernst M (2016). Targeting JAK kinase in solid tumors: emerging opportunities and challenges. Oncogene.

[16] Barcellos-Hoff MH, Lyden D, Wang TC (2013). The evolution of the cancer niche during multistage carcinogenesis. Nat Rev Cancer.

[17] Peng CW, Wang LW, Fang M (2013). Combined features based on MT1-MMP expression, CD11b+ immunocytes density and LNR predict clinical outcomes of gastric cancer. J Transl Med..

[18] Peng CW, Liu X-L, Chen C (2011). Patterns of cancer invasion revealed by QDs-based quantitative multiplexed imaging of tumor microenvironment. Biomaterials.

[19] Huijbers A, Tollenaar RA, Pelt GWV (2013). The proportion of tumor-stroma as a strong prognosticator for stage II and III colon cancer patients: validation in the VICTOR trial. Ann Oncol.

[20] Mesker WE, Junggeburt JM, Szuhai K (2007). The carcinoma-stromal ratio of colon carcinoma is an independent factor for survival compared to lymph node status and tumor stage. Cell Oncol..

[21] Liu J, Liu J, Li J (2014). Tumor-stroma ratio is an independent predictor for survival in early cervical carcinoma. Gynecol Oncol.

[22] Wang K, Ma W, Wang J (2012). Tumor-stroma ratio is an independent predictor for survival in esophageal squamous cell carcinoma. J Thorac Oncol..

[23] Moorman AM, Vink R, Heijmans HJ (2012). The prognostic value of tumour-stroma ratio in triple-negative breast cancer. Eur J Surg Oncol.

[24] Marchet A, Mocellin S, Ambrosi A (2007). The ratio between metastatic and examined lymph nodes (N ratio) is an independent prognostic factor in gastric cancer regardless of the type of lymphadenectomy: results from an Italian multicentric study in 1853 patients. Ann Surg.

[25] Association JGC (2011). Japanese classification of gastric carcinoma: 3rd English edition. Gastric Cancer.

[26] Teng MWL, Ngiow SF, Ribas A (2015). Classifying cancers based on T-cell infiltration and PD-L1. Cancer Res.

[27] Gentles AJ, Newman AM, Liu CL (2015). The prognostic landscape of genes and infiltrating immune cells across human cancers. Nat Med.

[28] Calon A, Lonardo E, Berenguer-Llergo A (2015). Stromal gene expression defines poor-prognosis subtypes in colorectal cancer. Nat Genet..

[29] Peng CW, Liu XL, Liu X (2010). Co-evolution of cancer microenvironment reveals distinctive patterns of gastric cancer invasion: laboratory evidence and clinical significance. J Transl Med..

[30] De Wever O, Mareel M (2003). Role of tissue stroma in cancer cell invasion. J Pathol..

[31] Kouniavsky G, Khaikin M, Zvibel I (2002). Stromal extracellular matrix reduces chemotherapy-induced apoptosis in colon cancer cell lines. Clin Exp Metastasis.

[32] Liotta LA, Rao CN, Barsky SH (1983). Tumor invasion and the extracellular matrix. Lab Invest.

[33] Catalano V, Turdo A, Di Franco S (2013). Tumor and its microenvironment: a synergistic interplay. Semin Cancer Biol.

[34] Lorusso G, Ruegg C (2008). The tumor microenvironment and its contribution to tumor evolution toward metastasis. Histochem Cell Biol.

[35] de Kruijf EM, van Nes JG, van de Velde CJ (2011). Tumor-stroma ratio in the primary tumor is a prognostic factor in early breast cancer patients, especially in triple-negative carcinoma patients. Breast Cancer Res Treat.

[36] Mesker WE, Liefers GJ, Junggeburt JM (2009). Presence of a high amount of stroma and downregulation of SMAD4 predict for worse survival for stage I–II colon cancer patients. Cell Oncol..

[37] Ueno H, Konishi T, Ishikawa Y (2014). Histologic categorization of fibrotic cancer stroma in the primary tumor is an independent prognostic index in resectable colorectal liver metastasis. Am J Surg Pathol.

[38] Ueno H, Shinto E, Shimazaki H (2015). Histologic categorization of desmoplastic reaction: its relevance to the colorectal cancer microenvironment and prognosis. Ann Surg Oncol.

