# Supplementary figures and images for: The tumor-stromal ratio as a strong prognosticator for advanced gastric cancer patients: proposal of a new TSNM staging system
Source: J Gastroenterol. 2017 Aug 16;53(5):606–17. doi: 10.1007/s00535-017-1379-1 (PMC5910462; doi:10.1007/s00535-017-1379-1)

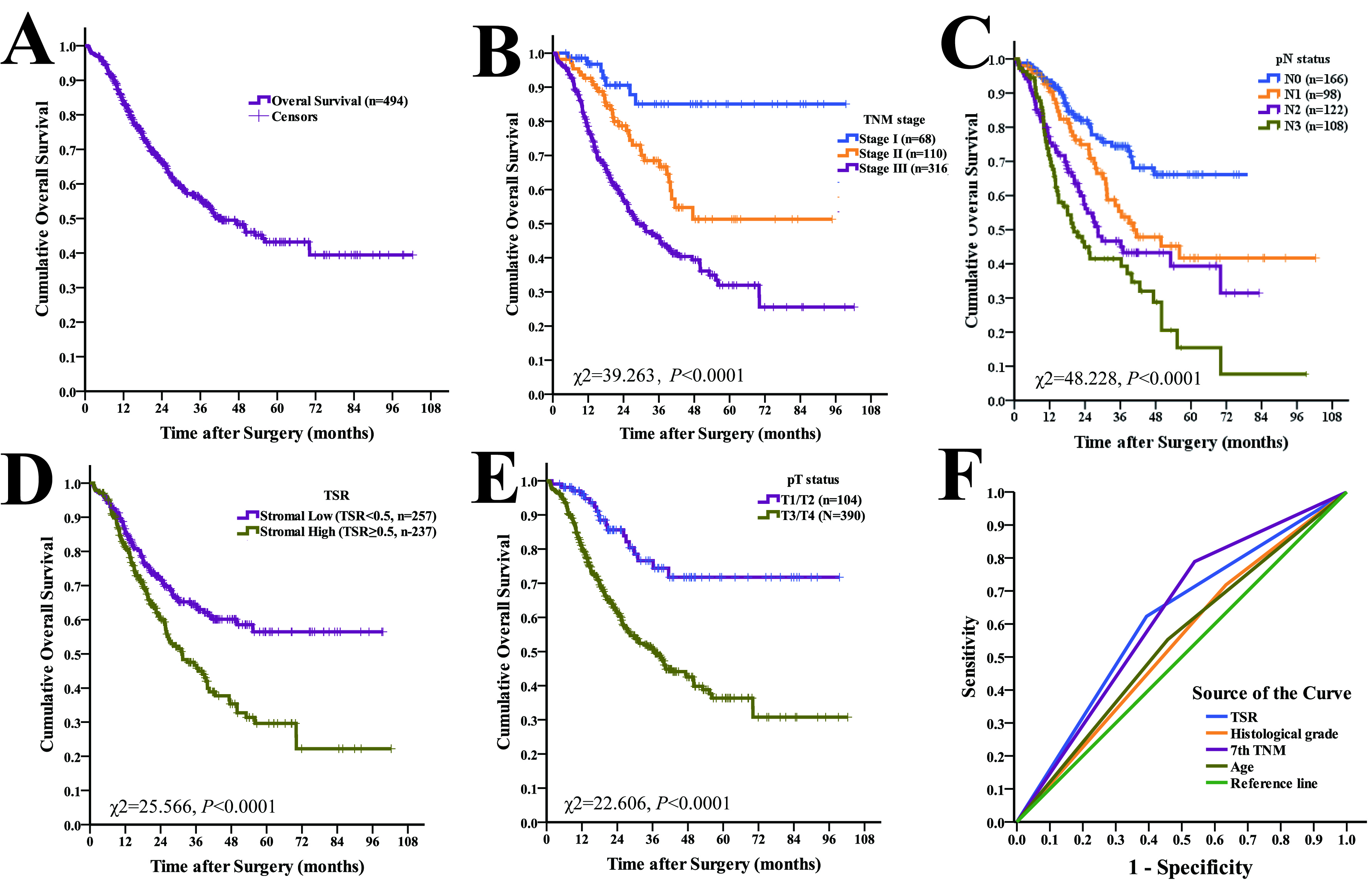

Supplement: Supplementary file 1 — Supplement Fig. 1. Cumulative OS of GC patients. (A) OS of 494 GC patients. (B) OS of all patients in TNM staging system. (C) Patients with lymph node metastasis was related to poor OS. (D) Patients in stromal-high group were as high risk for death. (E) Patients with serosa invasion was related to poor OS. (F) TSR performed well in predicting the clinical outcomes of GC patients compared to other factors. Area under the curve for TSR was 0.615 (95%CI: 0.584-0.665, P < 0.001). Area under the curve for histological grade was 0.542 (95%CI: 0.491-0.594, P = 0.110), Area under the curve for 7th TNM staging system was 0.625 (95%CI: 0.576-0.674, P < 0.001); Area under the curve for age was 0.528 (95%CI: 0.496-0.599, P = 0.073). (JPEG 3209 kb) [file 535_2017_1379_MOESM1_ESM.jpg]
