# Supplementary material for: The tumor-stromal ratio as a strong prognosticator for advanced gastric cancer patients: proposal of a new TSNM staging system
Source: J Gastroenterol. 2017 Aug 16;53(5):606–17. doi: 10.1007/s00535-017-1379-1 (PMC5910462; doi:10.1007/s00535-017-1379-1)
Supplement: Supplementary file 2 — Supplementary material 2 (DOCX 22 kb) [file 535_2017_1379_MOESM2_ESM.docx]

| **Supplement Table 1: Discrimination ability of TNM system and TSR regarding Overall Survival (OS)** | | | | | | |
| --- | --- | --- | --- | --- | --- | --- |
| **Variables** | **N** | **N of deaths  (%)** | **3-year survival rate** | **5-year survival rate** | **Log-rank test χ2 value** | ***P*-value ^a^** |
| Stage I | 68 | 7 (10.3) | 85.0% | 85.0% |  |  |
| IA/IB |  |  |  |  |  |  |
| IA | 18 | 0 (0) | 100% | 100% | 1.459 | 0.227 |
| IB | 51 | 7 (13.7) | 81.8% | 81.8% |  |  |
| TSR |  |  |  |  |  |  |
| S0 | 42 | 3 (7.1) | 90.6% | 90.6% | 2.384 | 0.123 |
| S1 | 27 | 4 (14.8) | 72.1% | 72.1% |  |  |
| Stage II | 110 | 35 (31.8) | 70.3% | 54.0% |  |  |
| IIA/IIB |  |  |  |  |  |  |
| IIA | 21 | 7 (33.3) | 59.7% | 44.7% | 0.426 | 0.514 |
| IIB | 89 | 26 (29.2) | 72.2% | 52.8% |  |  |
| TSR |  |  |  |  |  |  |
| S0 | 63 | 12 (19.0) | 81.6% | 61.4% | 11.090 | **0.001** |
| S1 | 47 | 21 (45.7) | 54.1% | 33.5% |  |  |
| Stage III | 316 | 157 (49.7) | 44.9% | 30.9 |  |  |
| IIIA/IIIB/IIIC |  |  |  |  |  |  |
| IIIA | 73 | 32 (43.8) | 57.9% | 43.7% | 16.643 | **<0.0001** |
| IIIB | 110 | 52 (47.3) | 42.8% | 35.6% |  |  |
| IIIC | 132 | 75 (56.8) | 39.9% | 19.2% |  |  |
| TSR |  |  |  |  |  |  |
| S0 | 149 | 60 (40.3) | 52.1% | 43.8 | 7.187 | **0.007** |
| S1 | 166 | 99 (59.6) | 38.9% | 21.5 |  |  |
| ^a:^ pS indicates the status of TSR. TSR is considered as a represent factor of stromal, and the S0 indicates stromal-low (TSR<0.5), the S1 indicates stromal-high (TSR≥0.5).  ^b^: Bold indicates values with a significant difference *P* < 0.05.  Abbreviations: OS: overall survival; TSR: tumor stromal ratio | | | | | | |
